# The association between night eating syndrome and health-related quality of life in Korean adults: a nationwide study

**DOI:** 10.1007/s40519-023-01532-9

**Published:** 2023-02-20

**Authors:** Woorim Kim, Yeong Jun Ju, Soon Young Lee

**Affiliations:** 1 Division of Cancer Control and Policy, National Cancer Control Institute, National Cancer Center, Goyang-si, Gyeonggi-do Republic of Korea; 2 Department of Preventive Medicine and Public Health, Ajou University School of Medicine, 206 World cup-ro, Yeongtong-gu, Suwon-si, Gyeonggi-do 16499 Republic of Korea

**Keywords:** Night eating syndrome, Feeding and eating disorders, Health-related quality of life, Quality of life, Sleep

## Abstract

**Purpose:**

Quality of life may be influenced by the presence of eating disorders. This study investigated the association between night eating syndrome (NES) and health-related quality of life in the general population.

**Methods:**

Data were from the 2019 Korea Community Health Survey. The presence of NES was determined using the Night Eating Questionnaire. Health-related quality of life was measured using the 3-level EuroQoL-5 Dimension Index. Multivariable linear regression analyses assessed the association between NES and health-related quality of life. Subgroup analyses were performed based on daily sleep duration.

**Results:**

A total of 34,434 individuals aged 19 years or older were included in the study population. Participants with NES (*β* = − 4.85, *p* < 0.001) reported poorer health-related quality of life scores than those without NES. Decreases in health-related quality of life scores among those with NES were greatest in those who slept over 8 h daily (*β* = − 12.03, *p* = 0.004), followed by those who slept less than 6 h (*β* = − 5.90, *p* = 0.006) and participants who slept between 6 and 8 h (*β* = − 3.40, *p* = 0.026) daily.

**Conclusion:**

Individuals with NES were more likely to have a lower health-related quality of life than those without NES. These findings highlight the potential importance of considering NES in investigating the health-related quality of life.

**Level of evidence:**

Level III, well-designed case–control analytic studies.

**Supplementary Information:**

The online version contains supplementary material available at 10.1007/s40519-023-01532-9.

## Introduction

Night eating syndrome (NES) is a non-normative eating pattern characterized by nocturnal eating, in which affected individuals consume most of their food during the evening and night [1, 2]. A few features proposed in the core criteria for NES include consuming at least 25 percent of daily calories after the evening meal, waking up at least twice per week to eat during the night, and individuals being aware of their food consumption [3]. Individuals with more severe NES symptoms report awakening and consuming nocturnal snacks [4]. NES has also been characterized in the Diagnostic and Statistical Manual of Mental Disorders (DSM-5) [5]. The prevalence of NES has been shown to rate between 1 and 1.5 percent in the general population [6].

Despite the fact that different types of eating disorders have been shown to significantly impact the quality of life (QoL) of individuals, in which people with symptoms of eating disorders exhibit noticeably lower QoL, relatively few studies investigated the relationship between NES and QoL [7]. Quality of life is a comprehensive concept referring to individuals’ satisfaction with their health and functioning across different aspects of life, including the physical, mental, cognitive, and social domains [8]. Specifically, health-related quality of life (HRQoL) encompasses areas of QoL that reflect an array of health status indices specific to physical, psychological, and social dimensions of health [9]. Considering that associations have been found between NES, poor physical and psychosocial functioning, and maladaptive coping, it is probable to suggest an association between NES and HRQoL [10].

The objective of this study was to investigate the association between NES and HRQoL using data from a large, nationally representative sample of South Korean adults. Additional subgroup analysis was conducted based on daily sleep duration (hours of sleep per day) because sleep duration can be an essential factor in the development of various diseases, with short or excessive sleep being associated with individuals’ mental health [11, 12]. We hypothesized that individuals with NES would report poorer HRQoL scores than those without NES and that such tendencies will be magnified in participants with shortened or extended sleep hours.

## Methods

### Data source and study sample

Data from the 2019 Korea Community Health Survey (KCHS) were used for the analyses. The KCHS is a cross-sectional study conducted by the Korea Centers for Disease Control and Prevention (KCDC) that includes a large, nationally representative sample of the Korean population selected using a two-stage sampling method in which primary sampling units are first selected and followed by households through systematic sampling. In-person interviews were conducted in the 254 local districts of Korea, in which the target population of each area is about 900 residents aged 19 years or older. Further details regarding the KCHS have been previously published [13].

All variables were collected on the KCHS survey administered to the entire study sample nationwide. However, information on NES, measured using the Night Eating Questionnaire (NEQ), was collected only for residents of the Gyeonggi province. The NEQ was initially administered on a trial basis in the Gyeonggi province before it was implemented to investigate episodes of night eating nationwide. The Gyeonggi province was selected because it is the most populous region of Korea, physically surrounds the capital city Seoul, consists of both large, urban cities and rural regions, and is known for its demographically, socioeconomically, and geographically diverse population that largely resembles the characteristics of the entire Korean population. Thus, this study used information collected from this province in the KCHS.

**Fig. 1:**
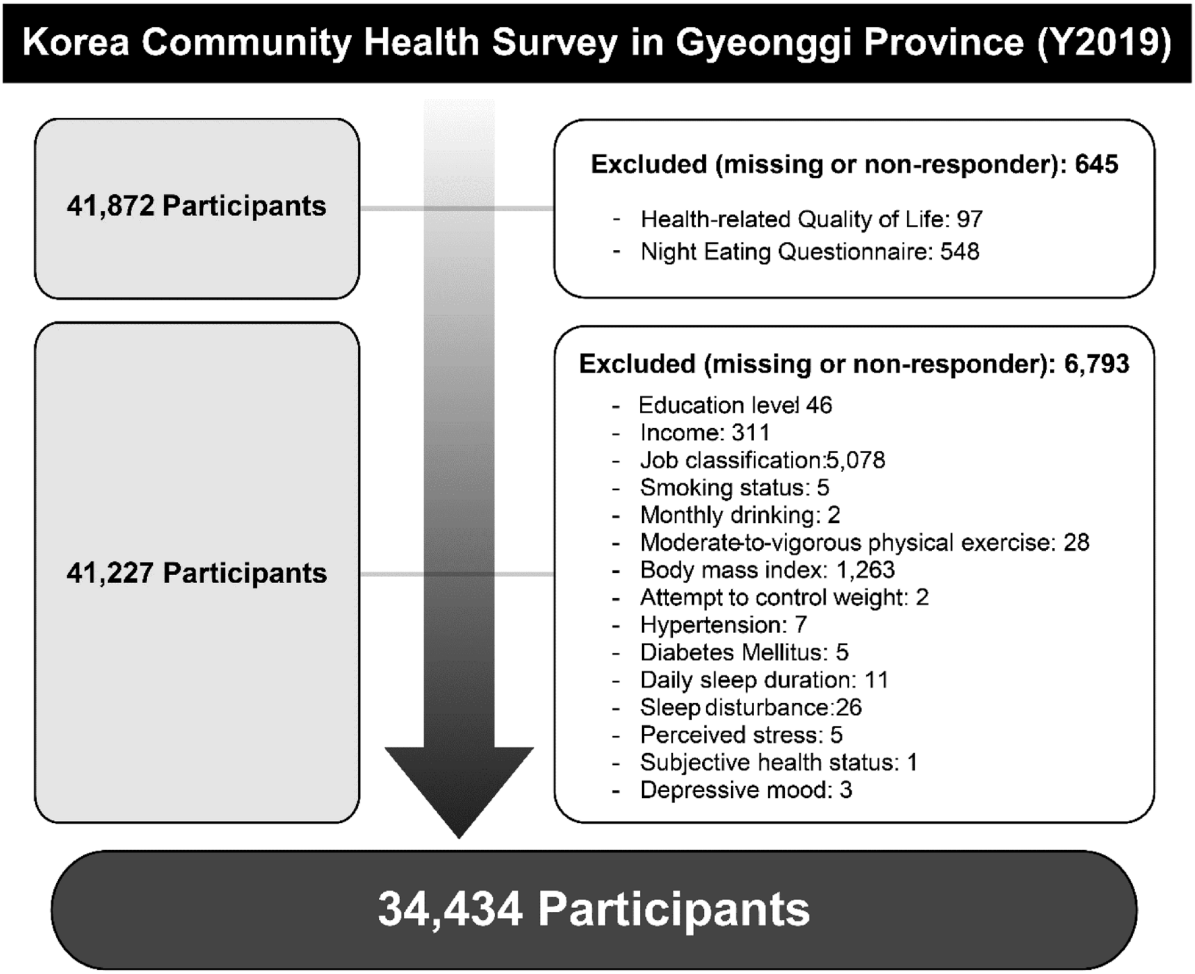
The study population selection process

As NES was the primary independent study variable, this study only included participants from the Gyeonggi province. Of the 41,972 participants initially identified, individuals with missing variables (or non-respondents) were excluded from the analyses. The final analytic sample consisted of 34,434 individuals aged 19 years or older. Details on the selection process of the study population can be found in Fig. 1.

### Dependent variable

The dependent variable was HRQoL, measured using the Korean version of the 3-level EQ-5D Index (EQ-5D-3L), introduced by the EuroQol Group, and has commonly been used to measure the quality of life [14]. The validity (Pearson correlation = 0.716, *p*-value < 0.001) and reliability (intraclass correlation coefficient = 0.652) of the index used have been verified [15]. This index is composed of five factors, namely mobility (M), self-care (SC), usual activities (UA), pain/discomfort (PD), and anxiety/depression (AD). Individuals are inquired whether they have no problems (level = 1), some problems (level = 2), or extreme problems (level = 3) for all the five components listed above. The formula to derive a composite score, published by the Korea Centers for Disease Control and Prevention is as follows [14]:

$${\text{EQ}} - {\text{5D}}\,{\text{index}}\, = \,{1}{-}\left( {0.0{5}\, + \,0.0{96}*{\text{M2}}\, + \,0.{418}*{\text{M3}}\, + \,0.0{46}*{\text{SC2}}\, + \,0.{136}*{\text{SC3}}\, + \,0.0{51}*{\text{UA2}}\, + \,0.{2}0{8}*{\text{UA3}}\, + \,0.0{37}*{\text{PD2}}\, + \,0.{151}*{\text{PD3}}\, + \,0.0{43}*{\text{AD2}}\, + \,0.{158}*{\text{AD3}}\, + \,0.0{5}*{\text{N3}}} \right)$$

In this index, if an individual has some problems (level = 2) in mobility (M), a value of ‘1’ is inserted into ‘M2’ in the formula above. If not, a value of ‘0’ is inserted instead. Similarly, if an individual has extreme problems (level = 3) in self-care (SC), a value of ‘1’ is inserted into ‘SC3’ in the formula above. If not, a value of ‘0’ is inserted. Likewise, this same method is applied for each of the components listed in the formula above to gain a composite score. Lastly, a score of “1” is inserted into N3 if an individual has reported having extreme problems for any of the five components listed above. The maximum score of the EQ-5D index is ‘1,’ which indicates the best health state [16]. The values have been multiplied by 100 for easier interpretation and visualization.

### Variable of interest

The variable of interest in the study was NES, which was measured using the Korean version of the NEQ. The NEQ is a 14-item questionnaire that is used to measure the behavioral and psychological symptoms of NES. Responses were rated on a 5-point Likert scale, and a cutoff score of 25 was used to indicate the presence of NES [17]. The validity and reliability of the Korean version of the NEQ have been previously confirmed [18], and NES has been successfully evaluated using this questionnaire in previous research [19].

### Covariates

The study used several demographic, socioeconomic, and health-related variables as covariates, including sex (male or female), age (19–29, 30–39, 40–49, 50–59, 60–69, or ≥ 70 years), education level (none, elementary school, middle school, high school, or college or above), income (quartiles), job type (professional or administrative position, office work, sales and service, agriculture and fishery, blue collar work or simple labor, or none), region (urban or rural), smoking (no or yes), monthly alcohol use (no or yes), moderate-to-vigorous physical exercise (no or yes), body mass index (BMI; underweight, normal, or obese), attempt to control body weight (no or yes), hypertension (no or yes), diabetes mellitus (no or yes), daily sleep duration (less than 6 h per day, 6 to 8 h per day, or over 8 h per day), sleep disturbance (no or yes), perceived stress (no or yes), subjective health status (no or yes), and depressive mood (no or yes). BMI was categorized into underweight (BMI < 18.5), normal weight (18.5 ≤ BMI < 25.0), and obesity (BMI ≥ 25.0). Daily sleep duration was measured using an open-ended question, “How many hours do you sleep on average?” Daily sleep duration was categorized as < 6 h, 6 to 8 h, and > 8 h per day based on previous research [20, 21]. Short sleepers included participants who slept less than 6 h per day and long sleepers who slept above 8 h per day [20]. Sleep disturbance was measured based on the question, “Did you experience difficulties in falling or staying asleep or sleeping too much in the past 2 weeks?”

### Statistical analysis

T-tests and ANOVAs were used to measure the general characteristics of the study population. The participants’ mean characteristics and HRQoL scores by their sociodemographic, economic, and health-related characteristics were calculated. Cohen’s *d* and partial eta-squared (η^2^), an effect-size measure, was calculated. These values estimate the degree of association for the sample. For Cohen’s *d,* small, medium, and large effect sizes are 0.2, 0.5, and ≥ 0.8, whereas 0.01, 0.06, and ≥ 0.14 are suggested for partial eta-squared [22]. Cohen’s d is calculated as Cohen’s d = $\frac{{M}_{1} - {M}_{2}}{{S}_{pooled}}$, where Spooled = $\sqrt{\frac{\left({n}_{1}-1\right){S}_{1}^{ 2} + ({n}_{2}-1){S}_{2}^{ 2}}{{n}_{1}+ {n}_{2}-2}}$. M1, S1, and n1 are the mean, standard deviation, and sample size of Group 1. M2, S2, and n2 represent the same measures for Group 2. In addition, partial eta-squared is calculated as η^2^partial = $\frac{{SS}_{effect}}{ {SS}_{effect}+ {SS}_{error}}$, where SSeffect and SSerror represent the sum of squares of effect and the sum of squared of error, respectively. Multivariable linear regression analyses assessed the association between NES and HRQoL. A subgroup analysis was conducted based on daily sleep duration. All analyses were performed after adjusting for covariates. All analyses were two-tailed and statistical significance was set at *p* < 0.05. Analyses were conducted using the SAS 9.4 software (SAS Institute, Cary, NC, USA).

### Ethical considerations

This study was conducted in accordance with the Declaration of Helsinki. The KCHS used in the analyses were publicly available data which had been deidentified and fully anonymized before its release. This study was included on the review list pursuant to Article 2.2 of the Enforcement Rule of Bioethics and Safety Act in Korea, since the data used were exempted from IRB review.

## Results

The general characteristics of the study population are shown in Table 1, along with the effect size by Cohen’s *d* and partial eta-squared. The mean HRQoL score for the overall sample of 34,434 individuals aged 19 years or above was 94.69 (SD = 9.78). A total of 197 (0.6%) participants reported having NES. Individuals with NES had lower mean HRQoL scores (M = 83.13, SD = 16.87) than those who did not have scores ≥ 25 on the NEQ (M = 94.75, SD = 9.68). Large effect sizes were found for differences in HRQoL scores between participants without and with episodes of night eating (*d* = 1.19, 95% CI = 1.053–1.334*)*. Decreased HRQoL scores were also found in participants who slept less than 6 h (M = 91.27, SD = 12.70) or above 8 h per day (M = 91.82, SD = 13.57) compared to those who slept 6 to 8 h per day (M = 95.51, SD = 8.67).

**Table 1:** General characteristics of subjects

Variables	Total		HRQoL*	*P* Value	Effect Size (95% CI)**	
	*N*	%	Mean ± S. D		Cohen's *d*	Partial η^2^
**Night eating syndrome**						
No (NEQ < 25)	34,237	99.4	94.75** ± **9.68	< 0.001	1.19 (1.053, 1.334)	−
Yes (NEQ ≥ 25)	197	0.6	83.13** ± **16.87			
**Sex**						
Male	16,705	48.5	96.09** ± **8.38	< 0.001	0.28 (0.260, 0.302)	−
Female	17,729	51.5	93.37** ± **10.77			
**Age**						
19–29	5490	15.9	97.79** ± **5.45	< 0.001	−	0.16 (0.151, 0.165)
30–39	4984	14.5	97.28** ± **5.78			
40–49	6615	19.2	96.91** ± **6.08			
50–59	6948	20.2	95.60** ± **8.33			
60–69	5267	15.3	93.47** ± **10.45			
70 +	5130	14.9	85.99** ± **14.92			
**Educational level**						
None	1498	4.3	81.16** ± **16.34	< 0.001	−	0.16 (0.150, 0.170)
Elementary school	3011	8.7	87.89** ± **13.97			
Middle school	2997	8.7	92.01** ± **11.71			
High school	13,322	38.7	95.97** ± **8.01			
College or above	13,606	39.5	97.02** ± **6.12			
**Income**						
Q1 (Low)	8583	24.9	89.89** ± **13.62	< 0.001	−	0.08 (0.078, 0.089)
Q2	7183	20.9	95.30** ± **8.60			
Q3	9899	28.7	96.45** ± **7.23			
Q4 (High)	8769	25.5	96.90** ± **6.58			
**Job classification**						
Professional or administrative position	5164	15.0	97.29** ± **5.81	< 0.001	−	0.06 (0.054, 0.064)
Office work	4007	11.6	97.29** ± **6.11			
Sales and service	4988	14.5	96.19** ± **7.08			
Agriculture and fishery	717	2.1	92.75** ± **10.55			
Blue collar work or simple labor	6666	19.4	95.86** ± **7.69			
Unemployed	12,892	37.4	91.77** ± **12.61			
**Region**						
Urban	27,022	78.5	95.08** ± **9.27	< 0.001	0.19 (0.161, 0.212)	
Rural	7412	21.5	93.26** ± **11.33			
**Smoking**						
No	27,955	81.2	94.42** ± **10.03	< 0.001	− 0.15 (− 0.176, − 0.121)	
Yes	6479	18.8	95.87** ± **8.51			−
**Monthly drinking**						
No	15,077	43.8	92.33** ± **11.96	< 0.001	− 0.44 (− 0.461, − 0.418)	
Yes	19,357	56.2	96.53** ± **7.14			−
**Moderate-to-vigorous physical exercise**						
No	26,860	78.0	94.23** ± **10.37	< 0.001	− 0.22 (− 0.242, − 0.191)	
Yes	7574	22.0	96.34** ± **7.09			−
**Body mass index**						
Underweight (BMI < 18.5)	1583	4.6	94.64** ± **10.33	< 0.001		0.00 (0.003, 0.005)
Normal (18.5 ≤ BMI < 25.0)	20,989	61.0	95.16** ± **9.20			
Obese (BMI ≥ 25.0)	11,862	34.4	93.87** ± **10.62			
**Attempt to control body weight**						
No	12,658	36.8	93.31** ± **11.51	< 0.001	− 0.22 (− 0.246, − 0.202)	
Yes	21,776	63.2	95.49** ± **8.51			
**Hypertension**						
No	26,545	77.1	95.90** ± **8.19	< 0.001	0.56 (0.531, 0.582)	
Yes	7889	22.9	90.60** ± **13.04			−
**Diabetes mellitus**						
No	31,197	90.6	95.22** ± **9.09	< 0.001	0.58 (0.548, 0.621)	
Yes	3237	9.4	89.58** ± **13.90			−
**Daily sleep duration**						
< 6 h	5710	16.6	91.27** ± **12.70	< 0.001		0.03 (0.025, 0.032)
6–8 h	27,615	80.2	95.51** ± **8.67			−
> 8 h	1109	3.2	91.82** ± **13.57			
**Sleep disturbance**						
No	24,128	70.1	96.57** ± **7.43	< 0.001	0.67 (0.648, 0.695)	
Yes	10,306	29.9	90.29** ± **12.76			
**Perceived stress**						
No	25,792	74.9	95.56** ± **8.75	< 0.001	0.36 (0.336, 0.385)	
Yes	8642	25.1	92.08** ± **11.97			
**Subjective health status**						
Poor	21,522	62.5	92.72 ± 11.28	< 0.001	− 0.56 (− 0.580, − 0.536)	
Fair	12,912	37.5	97.99 ± 5.06			−
**Depressive mood**						
No	32,098	93.2	95.43 ± 8.76	< 0.001	1.15 (1.111, 1.197)	
Yes	2336	6.8	84.59 ± 15.70			−
**Total**	34,434	100.0	94.69 ± 9.78			

*Health-related Quality of Life

**Effect size was measured using Cohen’s d for *t*-test and partial eta-squared (η^2^) for ANOVA analyses. For Cohen’s d, the categorical values for small, medium, and large effect sizes are 0.2, 0.5, and ≥ 0.8, whereas 0.01, 0.06, and ≥ 0.14 are suggested for partial eta-squared

The results of the multivariable regression analysis on the association between HRQoL and NES are presented in Table 2. Compared to individuals without NES, those with NES had significantly lower HRQoL scores (β = − 4.85, *p* < 0.001). Regarding daily sleep duration, participants who reported sleeping less than 6 h per day (β = − 0.65, *p* < 0.001) had lower HRQoL scores than those reporting 6 to 8 h of sleep, in addition to those reporting sleeping over 8 h per day (β = − 0.75, *p* = 0.025).

**Table 2:** The association between health-related quality of life (HRQoL) and night eating syndrome

Variables	HRQoL		
	Adjusted-β*	S.E	*P* value
**Night eating syndrome**			
No (NEQ < 25)	Ref		
Yes (NES ≥ 25)	− 4.85	1.25	< 0.001
**Sex**			
Male	Ref		
Female	− 0.77	0.11	< 0.001
**Age**			
19–29	Ref		
30–39	− 0.22	0.12	0.065
40–49	− 0.67	0.12	< 0.001
50–59	− 1.09	0.14	< 0.001
60–69	− 1.31	0.19	< 0.001
70 +	− 5.03	0.30	< 0.001
**Educational level**			
None	Ref		
Elementary school	3.94	0.56	< 0.001
Middle school	6.15	0.55	< 0.001
High school	7.52	0.54	< 0.001
College or above	7.65	0.54	< 0.001
**Income**			
Q1 (low)	Ref		
Q2	1.00	0.17	< 0.001
Q3	1.11	0.16	< 0.001
Q4 (high)	1.19	0.16	< 0.001
**Job classification**			
Professional or administrative position	Ref		
Office work	− 0.31	0.12	0.010
Sales and service	− 0.13	0.14	0.332
Agriculture and fishery	− 0.40	0.47	0.387
Blue collar work or simple labor	0.09	0.13	0.475
Unemployed	− 1.20	0.13	< 0.001
**Region**			
Urban	Ref		
Rural	0.00	0.14	0.977
**Smoking**			
No	Ref		
Yes	0.00	0.12	0.978
**Monthly drinking**			
No	Ref		
Yes	1.02	0.10	< 0.001
**Moderate-to-vigorous physical exercise**			
No	Ref		
Yes	0.32	0.09	0.001
**Body mass index**			
Underweight (BMI < 18.5)	Ref		
Normal (18.5 ≤ BMI < 25.0)	0.10	0.26	0.697
Obese (BMI ≥ 25.0)	− 0.42	0.27	0.121
**Attempt to control body weight**			
No	Ref		
Yes	0.57	0.10	< 0.001
**Hypertension**			
No	Ref		
Yes	− 0.53	0.16	0.001
**Diabetes mellitus**			
No	Ref		
Yes	− 1.03	0.25	< 0.001
**Daily sleep duration**			
6–8 h	Ref		
< 6 h	− 0.65	0.14	< 0.001
> 8 h	− 0.75	0.33	0.025
**Sleep disturbance**			
No	Ref		
Yes	− 2.84	0.12	< 0.001
**Perceived stress**			
No	Ref		
Yes	− 2.04	0.12	< 0.001
**Subjective health status**			
Poor	Ref		
Fair	1.87	0.08	< 0.001
**Depressive mood**			
No	Ref		
Yes	− 6.30	0.31	< 0.001

*Adjusted for sex, age, education level, income, job classification, region, smoking status, monthly drinking status, moderate-to-vigorous physical exercise, body mass index, attempt to control body weight, hypertension, diabetes mellitus, daily sleep duration, sleep disturbance, perceived stress, subjective health status, and depressive mood

The results of the subgroup analysis conducted based on daily sleep duration can be found in Table 3. The tendencies of the main findings were generally maintained regardless of daily sleep duration, in which the analysis showed marginal significance (p-value = 0.1303). However, the decreases in HRQoL scores found in individuals with NES were most profound in the group of participants who reported sleeping over 8 h (β = − 12.03, *p* = 0.004), followed by the group reporting sleeping less than 6 h (β = − 5.90, *p* = 0.006), and the group sleeping 6 to 8 h per day (β = − 3.40, *p* = 0.026).

**Table 3:** Results of the subgroup analysis

Variables		HRQoL		
		Adjusted-β*	S.E	*P* value
Daily sleep duration	Night eating syndrome			
< 6 h	No (NEQ < 25)	Ref		
	Yes (NES ≥ 25)	− 5.90	2.13	0.006
6–8 h	No (NEQ < 25)	Ref		
	Yes (NES ≥ 25)	− 3.40	1.52	0.026
> 8 h	No (NEQ < 25)	Ref		
	Yes (NES ≥ 25)	− 12.03	2.56	0.004

*Adjusted for sex, age, education level, income, job classification, region, smoking status, monthly drinking status, moderate-to-vigorous physical exercise, body mass index, attempt to control body weight, hypertension, diabetes mellitus, sleep disturbance, perceived stress, subjective health status, and depressive mood

## Discussion

This study investigated the association between NES and HRQoL using data from a large, nationally representative sample of Korean adults. Poorer HRQoL was found among individuals who scored 25 points or more on the NEQ, which indicates the presence of NES. Large effect sizes were found for differences in PHQ-9 scores between participants with and without episodes of night eating. In addition, decreases in HRQoL scores found among participants with symptoms of NES were particularly profound in individuals who reported short (less than 6 h) and long (over 8 h) daily sleep hours.

The findings add new information regarding the association between the presence of NES and poorer HRQoL in the general population. Although this relationship has received relatively less research, several previous studies have explored the potential relationship between HRQoL and eating disorders, including anorexia nervosa, bulimia nervosa, binge eating disorder, and other specified feeding and eating disorders [8]. Past literature has generally suggested that HRQoL is poorer in individuals with eating disorders, with some studies indicating long-term impairments [23]. Specifically, a study with a sample of Italian women revealed that patients with eating disorders report decreased HRQoL, regardless of whether they were inpatients or outpatients [24]. A systematic review confirmed that eating disorders could have a noticeable impact on HRQoL, and patients with anorexia nervosa, bulimia nervosa, and binge eating disorder reported significantly worse scores than the healthy comparison groups [25]. Similarly, a previous study theorized that individuals with NES may have poorer quality of life since NES is known to increase the risk of many disorders, including affective disorders [10, 26]. Since HRQoL encompasses a diverse spectrum of health-related functioning, poorer HRQoL scores may be found in individuals with NES as revealed in this study.

The correlation between NES and HRQoL scores found in this study was present regardless of the number of hours slept daily. Still, the current findings show that the magnitude of this relationship is greater among those with long sleep duration (over 8 h per day), followed by those with short sleep duration (less than 6 h per day). This tendency may be partially explained by the fact that impaired sleep and oversleeping can exert various health effects, such as increasing the risk of developing different chronic diseases and increased mortality [27]. A study on Japanese adults revealed that short sleep duration has been associated with poorer physical and mental quality of life [28]. Excessive sleep was also found as a marker of poorer quality of life and HRQoL in the general population and the elderly [29, 30]. Considering the potential negative impact of impaired or excessive sleep on quality of life, individuals with long or short daily sleep hours may have experienced larger decreases in HRQoL according to the presence of NES than those who received the recommended hours of sleep.

### Strength and limitations

This study has some limitations. First, as this study had a cross-sectional design, any interpretations that infer causality should be made with caution. Second, only residents of the Gyeonggi province were included in this study because the NEQ variable was inquired on this subset of the study population in the 2019 KCHS survey. However, the findings can be generalized to an extent to other regions because the Gyeonggi province is the most populous region of Korea and includes a diverse population that resides in both urban and rural areas. Third, the analytic results could have been underestimated since ceiling effects have been reported for the EQ-5D index. Last, daily sleep duration was measured using self-reports in the KCHS. Despite such limitations, this study is unique because it is the first to analyze the association between NES and HRQoL in the general population of Korea.

## Conclusions

This study showed that individuals with NES had significantly lower HRQoL than those without NES. The association between NES and HRQoL was particularly notable among individuals who had short or long daily sleep hours. The findings suggest the potential importance of monitoring and accounting for NES in addressing the HRQoL of the general population.

## What is already known on this subject?

Previous studies have reported an association between health-related quality of life and various eating disorders. Yet few studies have focused on night eating syndrome, a disorder characterized by nocturnal eating in which affected individuals consume most of their food during the evening and night.

## What this study adds?

A significant association was found between night eating syndrome and health-related quality of life, in which individuals with symptoms of night eating syndrome showed poorer health-related quality of life. Such tendencies were magnified in individuals who had short (less than 6 h) or long (above 8 h) daily sleep hours.

## Supplementary Information

Below is the link to the electronic supplementary material.

Supplementary file1 (DOCX 16 KB)

## Data Availability

Data will be made available on request. The dataset is available on the Korea Community Health Survey website (https://chs.kcda.go.kr/chs/rdr/rdrInfoProcessMain.do).
