# Supplementary material for: The association between night eating syndrome and health-related quality of life in Korean adults: a nationwide study
Source: Eat Weight Disord. 2023 Feb 20;28(1):17. doi: 10.1007/s40519-023-01532-9 (PMC9941275; doi:10.1007/s40519-023-01532-9)
Supplement: Supplementary file 1 — Supplementary file1 (DOCX 16 KB) [file 40519_2023_1532_MOESM1_ESM.docx]

**Supplementary Table S1. Mean Night Eating Questionnaire (NEQ) scores according to the five domains of the EQ-5D Index**

| **Variables** | **NEQ scores** | | | | |
| --- | --- | --- | --- | --- | --- |
|  | **Mean** | **±** | | **S. D** | |
| **Mobility** |  | |  | |  |
| No problems | 9.45 | | **±** | | 4.44 |
| Moderate/extreme | 10.65 | | **±** | | 5.31 |
| p-value | <.001 | | | | |
| **Self-care** |  |  |  |  |  |
| No problems | 9.50 | | **±** | | 4.49 |
| Moderate/extreme | 11.74 | | **±** | | 5.66 |
| p-value | <.001 | | | | |
| **Usual activities** |  | |  | |  |
| No problems | 9.46 | | **±** | | 4.45 |
| Moderate/extreme | 11.09 | | **±** | | 5.47 |
| p-value | <.001 | | | | |
| **Pain/discomfort** |  | |  | |  |
| No problems | 9.18 | | **±** | | 4.30 |
| Moderate/extreme | 10.63 | | **±** | | 5.00 |
| p-value | <.001 | | | | |
| **Anxiety/depression** |  | |  | |  |
| No problems | 8.85 | | **±** | | 4.04 |
| Moderate/extreme | 13.64 | | **±** | | 5.08 |
| p-value | <.001 | | | | |
